# High abundance synovial fluid proteome: distinct profiles in health and osteoarthritis

**DOI:** 10.1186/ar2172

**Published:** 2007-04-02

**Authors:** Reuben Gobezie, Alvin Kho, Bryan Krastins, David A Sarracino, Thomas S Thornhill, Michael Chase, Peter J Millett, David M Lee

**Affiliations:** 1 The Case Center for Proteomics, Case Western Reserve University School of Medicine, Euclid Avenue, Cleveland, Ohio 44106, USA

## Abstract

The development of increasingly high-throughput and sensitive mass spectroscopy-based proteomic techniques provides new opportunities to examine the physiology and pathophysiology of many biologic fluids and tissues. The purpose of this study was to determine protein expression profiles of high-abundance synovial fluid (SF) proteins in health and in the prevalent joint disease osteoarthritis (OA). A cross-sectional study of 62 patients with early OA (*n *= 21), patients with late OA (*n *= 21), and control individuals (*n *= 20) was conducted. SF proteins were separated by using one-dimensional PAGE, and the in-gel digested proteins were analyzed by electrospray ionization tandem mass spectrometry. A total of 362 spots were examined and 135 high-abundance SF proteins were identified as being expressed across all three study cohorts. A total of 135 SF proteins were identified. Eighteen proteins were found to be significantly differentially expressed between control individuals and OA patients. Two subsets of OA that are not dependent on disease duration were identified using unsupervised analysis of the data. Several novel SF proteins were also identified. Our analyses demonstrate no disease duration-dependent differences in abundant protein composition of SF in OA, and we clearly identified two previously unappreciated yet distinct subsets of protein profiles in this disease cohort. Additionally, our findings reveal novel abundant protein species in healthy SF whose functional contribution to SF physiology was not previously recognized. Finally, our studies identify candidate biomarkers for OA with potential for use as highly sensitive and specific tests for diagnostic purposes or for evaluating therapeutic response.

## Introduction

Osteoarthritis (OA), which is characterized by progressive destruction of articular cartilage, is by far the most common musculoskeletal disorder in the world, afflicting 40 million people in the USA alone [1,2]. Although this disorder is one of the most common among the aging population, our understanding of its etiology and pathophysiology, as well as our ability to detect early disease, is strikingly poor. A number of factors have frustrated efforts to elucidate the disease, and to develop diagnostic and treatment approaches; these include conflicting observations in epidemiologic studies, protracted disease duration, poorly correlated symptoms and radiographic findings, and lack of effective therapies. Compounding these difficulties, experimental mouse models are lacking and diseased tissue for experimental analyses is typically obtained from patients with advanced disease at joint replacement surgery, thereby limiting insight to late stages of disease.

These challenges notwithstanding, extensive disease-focused research has revealed that OA is not simply the result of age-related cartilage wear. Rather, the pathophysiology of disease involves the entire joint structure, including cartilage, synovium, ligaments, subchondral bone, and periarticular muscle. Documented contributors to this pathophysiology include genetic predisposition, trauma, inflammation, and metabolic changes. These insights have led many authorities to hypothesize that OA is best thought of as a group of disorders with varied etiologies whose final common clinical phenotypes converge [3].

There exists a particular dearth of understanding of etiologic contributors in early OA pathophysiology and stage-specific events in disease progression. Because synovial fluid (SF) is in contact with the primary tissues affected by disease (cartilage and synovium) and has been implicated as a contributor to disease pathophysiology, we hypothesized that proteomic analysis of SF may provide a minimally invasive opportunity to derive further stage-specific insight into OA disease. The advent of increasingly high-throughput and sensitive mass spectroscopy analytic methods and powerful statistical modeling, combined with exhaustive sequencing of the human genome, have facilitated unsupervised proteomic approaches to discovery of disease mechanisms. Here, we report on the results of a pilot cross-sectional study utilizing liquid chromatography with tandem mass spectrometry (LC-MS/MS) designed to identify differential expression of high-abundance SF proteins from healthy individuals and patients with early-stage and late-stage OA. Our analyses define a relative abundance of a large number of SF proteins and demonstrate that the protein composition of SF differs substantially between healthy individuals and patients with OA. Interestingly, although our data suggest that there is no significant change in the composition of high-abundance proteins between early and late OA, we identify distinct patterns of protein expression within OA patients that suggests identifiable subsets of disease that are independent of disease duration. Furthermore, we identify a panel of protein biomarkers that are of potential use in distinguishing SF from patients with OA from that of healthy study participants.

## Materials and methods

The experimental design for this study involved differential protein profiling of knee SF, using LC-MS/MS, from 20 healthy control individuals and two cohorts of 21 patients diagnosed with early and late OA. All samples for the study were collected from patients within our tertiary care referral center. Our hospital's institutional review board approved all aspects of this study. All SF samples included in the study were snap-frozen in liquid nitrogen immediately after acquisition from the knee joint.

### Control individuals

Twenty individuals without any prior history of knee trauma, chronic knee pain, prior knee surgery, blood dyscrasias, cancer, chondrocalcinosis, corticosteroid injection, or nonsteroidal anti-inflammatory drug use during the preceding eight weeks were recruited and underwent plain anterior-posterior, lateral, and sunrise view radiographs of their right/left knee. A total of 78 individuals qualified for entry into our study based on the criteria specified above and formed the study 'control' cohort. An arthrocentesis was attempted on each of these patients in order to obtain the 20 samples required for our study design. Samples that were free from visible blood contamination and consisted of a minimum of 500 μl were included in the study.

### Patients with early osteoarthritis

Samples were procured from 21 patients presenting for elective arthroscopic debridement of an inner-third tear of the medial meniscus with a minimum age of 45 years. Inner-third meniscal tears are relatively avascular, and therefore they are least likely to generate an inflammatory response that could confound proteomic analysis of protein expression related expressly to OA. No patients with a prior history of clinically significant knee trauma or infection, surgery, blood dyscrasia, cancer, corticosteroid injection, or chondrocalcinosis were included in the study. Because of the meniscal tear, prior nonsteroidal anti-inflammatory drug use was not a practical exclusion criterion. The diagnosis of early OA was made at the time of arthroscopy based on the presence of arthroscopically visible chondral erosion. SF was acquired at the time of arthroscopic trocar placement in order to avoid blood contamination of the samples.

### Patients with late osteoarthritis

One SF sample was procured from each of 21 patients presenting for elective total knee replacement for management of primary idiopathic OA. The exclusion criteria were identical to those for patients with early OA. Each patient had documented joint space narrowing of all three compartments of the knee on plain radiographs. The SF was acquired from the knee joint before arthrotomy so as to avoid blood contamination.

### Power analysis

Supervised pair-wise comparisons were performed for each protein between the three disease classes (control: *n *= 20; early OA: *n *= 21; and late OA: *n *= 18). Here, in the least optimal two-class comparison scenario, the two classes of sample size 18 (patients with late OA) and 20 (control individuals) possess a minimal statistical power of 80% at the 0.05 level of significance (α) to detect a 50% relative difference in the presence/abundance of a tested protein between classes. The null hypothesis was that there is no difference in the distribution of the tested protein's presence/abundance between the two classes.

### Reduction/alkylation of synovial fluid samples and electropheresis

Each sample was reduced and alkylated in a lysis buffer before it was subjected to electrophoresis. Each sample was fractionated into nine molecular weight regions. An in-gel tryptic digestion was performed on the nine slices from each sample. After 24 hours of tryptic digestion, the peptides were extracted and lyophilized to dryness. The lyophilate was re-dissolved into a loading buffer for mass spectrometry.

### Mass spectrometry

Samples are run on a LCQ DECA XP plus Proteome X workstation (Thermo-Finnigan, Waltham, MA, USA). For each run (2.5 hours), half of each sample was separated on a 75 μm (internal diameter) × 18 cm column packed with C18 media. In between each sample, a standard of a 5 Angio mix peptides was eluted (Michrom BioResources, Auburn, CA, USA) to ascertain column performance, and observe any potential carryover that might have occurred. The LCQ is run in a top five configuration, with one mass spectrometry scan and five tandem mass spectrometry scans.

### Processing of mass spectrometry data

Mass spectrometric peptide sequence spectra were searched against the National Center for Biotechnology Information's RefSeqHuman database [4] with the addition of contaminants using SEQUEST [5]. Variable modifications for oxidized methione and carboxyamidomethylated cysteine were permitted. Data were filtered using the following criteria: Xcorr greater than or equal to 1.5, 2.5 and 3.0 for a charge state of 1, 2 and 3, respectively; a ΔCn greater than 0.1; and an RSp equal to 1. All peptides satisfying these criteria were then mapped back to all human protein sequences in RefSeq, with a string search for exact matches. For each gene identified within a gel slice a minimal (duplicates removed) set of peptides was identified. This list was sorted by the total number of peptides in descending order. The first peptide array in this list was defined as a cluster and compared pair-wise with every other array in the list by determining whether the N-1 comparison was an equal or a proper subset. If the peptide array was found to be an equal or proper subset, then it was added to the cluster and removed from the list. The process was repeated until all comparisons were exhausted. For each cluster, the gene with the greatest number of peptide elements was assigned to designate the cluster. If multiple genes within the cluster had the same number of peptides, then an arbitrarily selected member was assigned as representative of the cluster. Peptides shared between clusters were identified and omitted from further analysis.

A total of 342 gel-slice distinct peptides were detected by LC-MS/MS in the 62 samples in this study. Each sample was divided into nine protein gel slices. These 342 slice-distinct peptides are comprised of 135 unique GenInfo accession-identified proteins. Peptide area was used as the primary measure of protein abundance in the study. Peptide area was calculated using the area function in BioWorks 3.1 (Thermo Electron Corporation, Waltham, MA, USA) with scan window of 60. Protein area was calculated as the sum of the areas for each independent analyte for all unique peptides within a protein cluster. If multiple areas were identified for a given analyte, then the largest area was selected and used in the in the area calculation. An independent analyte is defined as unique mass to charge identified in the SEQUEST search satisfying the filtering criteria.

### Principal component analysis

Recalling that 342 slice-distinct peptides were assayed throughout the 62 samples in this study, each sample is represented as a mathematical vector of 342 feature components. Each feature component is the area readout of a specific gel slice-distinct peptide indicating the abundance of that peptide in the sample. The primary dataset is a 342-peptide × 62-sample matrix of area readouts. Unsupervised principle component analysis (PCA) was used to assess the global sample variations and relationships in this dataset – between all 62 samples across 342 protein features – and to summarize the dataset in terms of a reduced number of dominant protein features that most affect the global sample variation [6-8]. Because we are using Pearson correlation as a measure of similarity between sample proteomic (area) profiles, each sample was normalized to have average 0 and variance 1 across its 342-feature protein areas before PCA. With area as a measure of slice-distinct protein abundance and sample profile similarity in terms of Pearson correlation, the first three principal components (PCs) capture 98.33% of global sample variation.

We note that the primary data matrix of 342 proteins × 62 samples is sparse; 13,628 (about 64%) of the 21,204 entries are 0, and the remaining non-zero area entries (about 36%) range from 10^1 ^to 10^6^. Given this characteristic of the data, two samples may have a high Pearson correlation that is due, artifactually, to a small number of outlying (extremal) area readouts. To mollify the effect of these outliers in global sample variations, we additionally performed PCA on sample-wise rank-normalized data. For each sample, the area of each peptide is replaced with the ranking of the peptide's area from 1 to 342 (or multiples of 1/2 within this range in cases where area values are identical) in relation to the areas of other peptides in that sample. Because we are using Pearson correlation as a measure of similarity between sample proteomic (area) profiles, each sample was normalized to have average 0 and variance 1 across its 342-feature peptide areas before PCA. With rank-normalized area as a measure of slice-distinct protein abundance and sample profile similarity in terms of Pearson correlation, the first three PCs capture 32.48% of global sample variation.

### Wilcoxon's rank sum test

For each protein, the nonparametric Wilcoxon's ranksum test was used to assess whether the difference in medians between two disease conditions (control, early OA, and late OA) of area measurements was statistically significant (whether the distributions of these area measurements overlap less than would be expected by chance) [9]. The null hypothesis is that the two independently measured conditions will be drawn from a single population, and therefore the medians will be equal. In this study, the null hypothesis (that a particular protein is differentially abundant) was rejected for *P *< 0.000001.

## Results

### Synovial fluid protein profiles

The proteins identified in our LC-MS/MS analyses are presented in Table 1. Note that 342 gel slice-distinct peptides, comprising a total cohort of 135 unique proteins, were detected across the 62 samples. Of these, 18 proteins represented keratin species (data not shown) that we considered to be contaminants from the cutaneous puncture performed during arthrocentesis and so removed them from further consideration, leaving a total of 117 SF proteins identified.

**Table 1:** Synovial fluid proteins identified

GI#	Protein
21493031	A kinase (PRKA) anchor protein 13
4501885	Actin, beta
4501887	Actin, gamma 1
4501889	Actin, gamma 2, smooth muscle, enteric
4501987	Afamin
6995994	Aggrecan 1 (chondroitin sulfate proteoglycan 1, large aggregating proteoglycan, antigen identified by monoclonal antibody A0122)
4502027	Albumin
55743106	Alpha 3 type VI collagen isoform 5 precursor (NP_476508)
21071030	Alpha-1-B glycoprotein
4502067	Alpha-1-microglobulin/bikunin precursor
4502337	Alpha-2-glycoprotein 1, zinc
4502005	Alpha-2-HS-glycoprotein
4557225	Alpha-2-macroglobulin
40254482	Amylase, alpha 1A; salivary
4502133	Amyloid P component, serum
4557287	Angiotensinogen (serine [or cysteine] proteinase inhibitor, clade A [alpha-1 antiproteinase, antitrypsin], member 8)
4502149	Apolipoprotein A-II
4502151	Apolipoprotein A-IV
4502153	Apolipoprotein B (including Ag [x] antigen)
4502157	Apolipoprotein C-I
32130518	Apolipoprotein C-II
4502163	Apolipoprotein D
4557325	Apolipoprotein E
4557327	Apolipoprotein H (beta-2-glycoprotein I)
4502397	B-factor, properdin
4757826	Beta-2-microglobulin
57634528	Carboxypeptidase N, polypeptide 2, 83 kD (NP_001300 removed for review)
47777317	Cartilage acidic protein 1
51944962	Cartilage intermediate layer protein (NP_003604)
40217843	Cartilage oligomeric matrix protein
4557485	Ceruloplasmin (ferroxidase)
42716297	Clusterin (complement lysis inhibitor, SP-40,40, sulfated glycoprotein 2, testosterone-repressed prostate message 2, apolipoprotein J)
42740907	Clusterin (complement lysis inhibitor, SP-40,40, sulfated glycoprotein 2, testosterone-repressed prostate message 2, apolipoprotein J)
4503635	Coagulation factor II (thrombin)
15011913	Collagen, type VI, alpha 1
7705753	Complement component 1, q subcomponent, alpha polypeptide
11038662	Complement component 1, q subcomponent, beta polypeptide
56786155	Complement component 1, q subcomponent, gamma polypeptide (NP_758957)
4502493	complement component 1, r subcomponent
41393602	Complement component 1, s subcomponent
14550407	Complement component 2
4557385	Complement component 3
4502501	Complement component 4A
14577919	Complement component 4A
50345296	Complement component 4B preproprotein (NP_001002029)
38016947	Complement component 5
4559406	Complement component 6
45580688	Complement component 7
4557393	Complement component 8, gamma polypeptide
54792787	Complement factor H-related 3 (NP_066303)
4885165	Cystatin A (stefin A)
4503107	Cystatin SA
42544239	D component of complement (adipsin)
16751921	Dermcidin
58530842	Desmoplakin isoform II (NP_001008844)
11761629	Fibrinogen, alpha chain isoform alpha preproprotein
4503689	Fibrinogen, alpha chain isoform alpha-E preproprotein
11761631	Fibrinogen, B beta polypeptide
4503715	Fibrinogen, gamma chain isoform gamma-A precursor
11761633	Fibrinogen, gamma chain isoform gamma-B precursor
47132557	Fibronectin 1 isoform 1 preproprotein
47132551	Fibronectin 1 isoform 2 preproprotein
16933542	Fibronectin 1 isoform 3 preproprotein
47132555	Fibronectin 1 isoform 4 preproprotein
47132553	Fibronectin 1 isoform 5 preproprotein
47132549	Fibronectin 1 isoform 6 preproprotein
4504165	Gelsolin (amyloidosis, Finnish type)
6006001	Glutathione peroxidase 3 (plasma)
32483410	Group-specific component (vitamin D binding protein)
4504375	H factor 1 (complement)
4826762	Haptoglobin
45580723	Haptoglobin-related protein
4504345	Hemoglobin, alpha 1
4504349	Hemoglobin, beta
4504351	Hemoglobin, delta
11321561	Hemopexin
4504489	Histidine-rich glycoprotein
4504579	I factor (complement)
21489959	Immunoglobulin J polypeptide, linker protein for immunoglobulin alpha and mu polypeptides
13399298	Immunoglobulin lambda-like polypeptide 1
4826772	Insulin-like growth factor binding protein, acid labile subunit
4504781	Inter-alpha (globulin) inhibitor H1
4504783	Inter-alpha (globulin) inhibitor H2
31542984	Inter-alpha (globulin) inhibitor H4 (plasma Kallikrein-sensitive glycoprotein)
4504893	Kininogen 1
54607120	Lactotransferrin (NP_002334)
4504985	Lectin, galactoside-binding, soluble, 7 (galectin 7)
5031885	Lipoprotein, Lp(a)
4505047	Lumican
9257232	Orosomucoid 1
4505529	Orosomucoid 2
19923106	Oaraoxonase 1
4505881	Plasminogen
51476111	PREDICTED: similar to Apolipoprotein A-I precursor (Apo-AI) (XP_496536)
51476113	PREDICTED: similar to Apolipoprotein C-III precursor (Apo-CIII) (XP_496537)
51472914	PREDICTED: similar to KIAA1501 protein (XP_370973)
4506355	Pregnancy-zone protein
4505821	Prolactin-induced protein
4506117	Protein S (alpha)
5031925	Proteoglycan 4, (megakaryocyte stimulating factor, articular superficial zone protein, camptodactyly, arthropathy, coxa vara, pericarditis syndrome)
55743122	Retinol-binding protein 4, plasma precursor (NP_006735)
4506773	S100 calcium binding protein A9 (calgranulin B)
50363217	Serine (or cysteine) proteinase inhibitor, clade A (alpha-1 antiproteinase, antitrypsin), member 1 (NP_000286)
50363221	Serine (or cysteine) proteinase inhibitor, clade A (alpha-1 antiproteinase, antitrypsin), member 1 (NP_001002235)
50363219	Serine (or cysteine) proteinase inhibitor, clade A (alpha-1 antiproteinase, antitrypsin), member 1 (NP_001002236)
4502595	Serine (or cysteine) proteinase inhibitor, clade A (alpha-1 antiproteinase, antitrypsin), member 6
50659080	Serine (or cysteine) proteinase inhibitor, clade A, member 3 precursor (NP_001076)
4502261	Serine (or cysteine) proteinase inhibitor, clade C (antithrombin), member 1
39725934	Serine (or cysteine) proteinase inhibitor, clade F (alpha-2 antiplasmin, pigment epithelium derived factor), member 1
4557379	Serine (or cysteine) proteinase inhibitor, clade G (C1 inhibitor), member 1 (angioedema, hereditary)
10835095	Serum amyloid A4, constitutive
41150478	Similar to immunoglobulin M chain
4507557	Tetranectin (plasminogen binding protein)
4557871	Transferrin
4507725	Transthyretin (prealbumin, amyloidosis type I)
4507895	Vimentin
18201911	Vitronectin (serum spreading factor, somatomedin B, complement S-protein)

GI#, GenInfo accession.

### Unsupervised principal component analysis of protein profiles

To identify variations between samples in terms of global SF proteomic profile, we used PCA of the 62 samples across the 342 slice-distinct protein area measurements. The initial PCA on the protein area measurements of all 62 samples identified three late OA sample profiles as statistical outliers from the remaining 59 samples (data not shown). These three outlier samples were removed from subsequent data analyses, leaving 342 slice-distinct proteins × 59 samples in the dataset under consideration. PCA of the protein area measurements of this dataset revealed that the two maximal and important directions of sample variance, PCs 1 and 2 accounted for 90.35% of the total sample variance. Notable, in the PC1-PC2 plane, control individuals (*n *= 20) are more homogeneous than patients with early OA (*n *= 21) and those with late OA (*n *= 19) in terms of global proteomic profile. The direction of maximal variance of PC1 correlates strongly with OA disease state.

**Figure 1:**
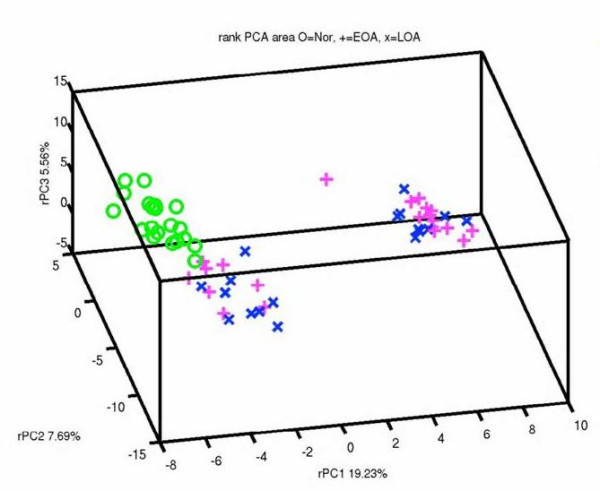
Principal component analysis of all 342 protein spots. Differential expression of the protein profile for healthy individuals versus patients with late and early osteoarthritis is observed using this unsupervised analytical technique. Note the two distinct subsets of protein expression in patients with osteoarthritis that cluster independently of disease duration. EOA, early osteoarthritis; LOA, late osteoarthritis; Nor, healthy individuals; PC, principal component; PCA, principal component analysis.

Next, the protein area measurements of this dataset of 342 slice-distinct proteins × 59 samples were rank-normalized for each sample (see Materials and methods, above) and PCA was performed on the resulting rank-normalized dataset. Similar to the protein area PCA, PCA of this dataset indicated that the control sample profiles were more homogeneous than the OA sample profiles (Figure 1). Although there was a clearer difference between the aontrol and OA sample profiles, this unsupervised analysis identified no definitive disease duration-dependent difference in expression profiles of SF high-abundance proteins in OA patients (Figure 1). Interestingly, despite this lack of difference between early-stage and late-stage disease, the PCA of the rank-normalized protein area profiles revealed two distinct subpopulations among OA samples, which we denote as OA group 1 (*n *= 17) and OA group 2 (*n *= 21). These two OA subpopulations do not appear to segregate by age, sex, ethnicity, or number of medications taken.

### Differentially abundant proteins in healthy versus osteoarthritis proteomic profiles

**Figure 2:**
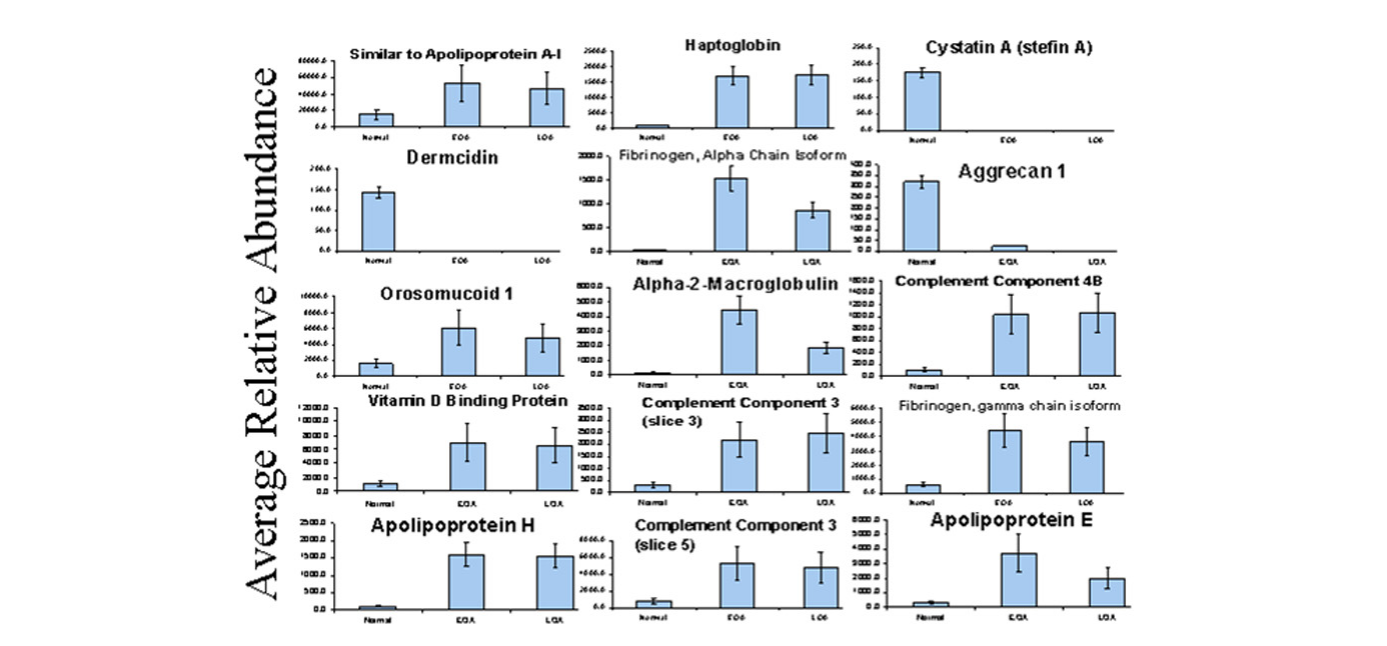
Relative quantitation of biomarkers using total ion current data from mass spectrometry. Determining cutoff values between control individuals and 'diseased' cohorts is among the necessary criteria in identifying protein or gene targets as 'biomarkers'. EOA, early osteoarthritis; LOA, late osteoarthritis.

We next sought to identify proteins that are differentially abundant (by area measures) between healthy individuals and patients with OA. Because the PCA analysis identified no significant difference between expression profiles from patients with early and late OA, we pooled data from these two cohorts and performed supervised Wilcoxon's ranksum tests to identify unique proteins with differential abundance between the healthy and OA groups. This method identified a subset of 18 of the 342 total proteins analysed that met our cutoff value for differential expression (*P *< 0.00001; Figure 2 and Table 2). The small *P *value used in this mathematical algorithm was chosen arbitrarily in order to reduce the number of candidate protein biomarkers identified to a manageable number that will appropriate for selective future study using more conventional techniques. Perhaps unsurprisingly, these 18 proteins are among the top 100 sample variation-contributing proteins in PC1 and PC2 in the previous PCA. Interestingly, a substantial majority (15/18) are significantly more abundant in the OA group than in the healthy group (Figure 2 and Table 2).

**Table 2:** Significant differentially abundant proteins identified

GI#	Protein description	Upregulated in	Specificity	Sensitivity	*P *value (Fisher's exact, two sided)
4885165	Cystatin A (stefin A)	Control	0.650	1.000	1.92 × e^-08^
6995994	Aggrecan 1 (chondroitin sulfate proteoglycan 1)	Control	0.650	0.974	2.30 × e^-07^
1651921	Dermcidin	Control	0.600	1.000	1.13 × e^-07^
4502027	Albumin	OA	0.950	0.718	7.96 × e^-07^
4502067	α_1_-Microglobulin/bikunin precursor	OA	0.950	0.718	7.96 × e^-07^
4503689	Fibrinogen, α chain isoform α-E preprotein	OA	0.950	0.718	7.96 × e^-07^
4503715	Fibrinogen, γ chain isoform γ-A precursor	OA	1.000	0.744	1.43 × e^-08^
4557225	α_2_-Macroglobulin	OA	0.950	0.718	7.96 × e^-07^
4557325	Apolipoprotein E	OA	1.000	0.744	1.43 × e^-08^
4557327	Apolipoprotein H (β_2_-glycoprotein I)	OA	1.000	0.744	1.43 × e^-08^
4557385	Complement component 3 (gel slice 3)	OA	0.950	0.718	7.96 × e^-07^
4557385	Complement component 3 (gel slice 5)	OA	1.000	0.744	1.43 × e^-08^
4557485	Ceruloplasmin (ferroxidase)	OA	0.950	0.718	7.96 × e^-07^
4826762	Haptoglobin	OA	0.950	0.718	7.96 × e^-07^
9257232	Orosomucoid 1	OA	0.850	0.667	2.51 × e^-04^
32483410	Group specific component (vitamin D binding protein)	OA	1.000	0.744	1.43 × e^-08^
50345296	Complement component 4B preprotein (NP_001002029)	OA	1.000	0.744	1.43 × e^-08^
5147611	PREDICTED: similar to apolipoprotein A-1 precursor (apo-A-1; XP_496536)	OA	0.950	0.718	7.96 × e^-07^
55743122	Retinol-binding protein 4, plasma precursor (NP_006735)	OA	0.900	0.692	1.87 × e^-05^

Eighteen proteins that were significantly differentially abundant (protein area) across control (*n *= 20) and OA (*n *= 39) groups by Wilcoxon's ranksum test at *P *< 1 × e^-06^. Sensitivity/specificity for each protein are calculated with respect to the number of samples of each group having protein area above or below the median area across all samples. The significance of median area dichotomy and true group label is assessed by two-sided Fisher's exact test. GI#, GenInfo accession.

### Differentially abundant proteins in discrete osteoarthritis subsets

**Figure 3:**
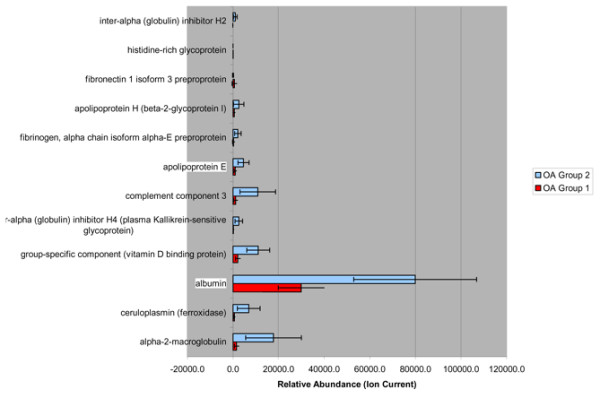
Proteins differentially expressed between two subtypes of osteoarthritis. OA, osteoarthritis.

Having identified two apparent subsets of patients with OA in our unsupervised PCA of the rank-normalized protein area data, we conducted a supervised Wilcoxon ranksum test to identify differential protein expression between these OA subsets irrespective of disease duration. Using a highly significant *P *value cutoff (*P *< 0.00005), we identified 12 proteins that exhibit differential expression between these OA subsets (Figure 3).

### Abundant synovial fluid proteins as potential biomarkers

Having identified a subset of 18 proteins with significantly different expression levels between patients with OA and healthy control individuals, we proceeded to explore the sensitivity and specificity of these proteins as biomarkers for differentiating health from disease. Examining sensitivity and specificity of individual proteins demonstrated that several of the 18 proteins in this panel hold promise as potential biomarkers for distinguishing health from disease (Figure 2 and Table 2). Indeed, the best sensitivity and specificity for proteins in this subset was noted for complement component 3, which exhibited sensitivity and specificity of 90% and 85%, respectively.

## Discussion

Although recent studies have highlighted the long appreciated importance of SF in joint function [10,11], identification of the protein constituents of SF and elucidation of their function remain areas of active investigation. Advances in proteomic analytic techniques afford new opportunities to gain insight into the function of complex biologic fluids in health and disease. By using one-dimensional gel electropheresis and LC-MS/MS, in the present study we provide quantitation of abundant proteins in SF in a cohort of 62 individuals, including healthy individuals and patients with early and late OA. Our results show clear differences in protein profiles between healthy and diseased SF, identify many SF proteins that are known to be involved in numerous homeostatic and pathologic pathways, and – intriguingly – identify two distinct subpopulations of patients with OA whose membership occurs independently of disease duration. These data coupled with the MudPIT (multidimensional protein identification technology) quantitation technique allows us to predict relative levels of protein expression within the context of a one-dimensional gel compared with a two-dimensional gel, as previously described [12-14].

Comparison of protein abundance between healthy individuals and OA patients identified 18 highly significant (*P *< 0.000001) and a large number of less statistically significant differentially expressed proteins (Tables 1 and 2, and Figure 2), many of which were previously identified by other investigators. Of these 18 proteins, three exhibit decreased expression levels in OA patients whereas 13 are more abundant in OA than in healthy individuals (Figure 2 and Table 2). This differential profile provides potential insight into the pathophysiology of OA. Increased abundance of aggrecan and cystatin A in SF from healthy individuals is consistent with the current concept that loss of cartilage observed in OA results from proteolytic destruction of extracellular matrix [15-24]. It is particularly interesting that cystatin A, an inhibitor of cysteine proteases (for example, cathepsins), is elevated in healthy SF, whereas serine protease inhibitors, which are abundant in health and disease in our analyses and have been implicated in the pathogenesis of OA [21,25-27], are not among the panel of highly significant differentially expressed proteins. This observation provides a strong rationale for continued focus on the contribution of both classes of protease inhibitors to OA pathogenesis.

Dermcidin, the third abundant SF protein demonstrating increased expression in normal individuals as compared with OA patients is a novel antimicrobial peptide that was previously identified in human sweat [28]. Dermcidin peptides exhibit broad-spectrum antimicrobial activity against bacteria and fungal species, and are derived from post-translational and post-secretion processing by a series of proteases that are present in sweat glands [28,29]. To our knowledge, this is the first report to identify dermcidin expression in SF; the role of this protein in healthy joint physiology and the pathophysiologic consequences of decreased expression in OA require further investigation.

Somewhat surprisingly, examination for disease stage-specific (early versus late OA) differences in abundant protein expression using unsupervised analyses revealed no significant differences in these cohorts. Although it is likely that further analysis of low-abundance proteins may yield stage-specific patterns of protein composition in SF, this finding is consistent with the hypothesis that subsets of pathogenic mechanisms that contribute to OA disease initiation are present throughout the course of disease. This observation holds significant promise for both early identification of patients at risk for subsequent severe OA and for early therapeutic interventions to interrupt progression of disease.

Intriguingly, our unsupervised analyses identify two clearly distinct subpopulations of patients with OA that are independent of disease duration. Supervised (Wilcoxon ranksum test) analysis identified 12 protein species differentially populating the SF of these OA subsets. It is noteworthy that proteins present in blood comprise the entire cohort of proteins that contribute to identification of these OA subpopulations. This observation could result from differences in vascular permeability as a distinguishing pathophysiologic feature of a disease subset in patients with OA. However, most of these proteins were identified more recently as products of the cells within joint tissue: chondrocytes and synoviocytes [30,31]. Thus, the differences observed could also reflect differences resulting from OA joint physiology. Unfortunately, the design of this pilot study precludes examination of phenotypic differences in these subgroups. Utilizing these 12 species in future expanded longitudinal cohorts of OA patients will further clarify both the presence of disease phenotype subsets and the utility of quantifying these proteins in SF as a method of identifying OA sub-phenotypes for prognostic and therapeutic purposes.

Although a primary objective of our study was examination of differential protein expression of abundant SF proteins between healthy individuals and OA patients, our analyses also provide a wealth of information about the abundant protein composition of SF in health. Many of the proteins identified have been implicated in pathways thought to contribute to the physiologic homeostasis of cartilage, synovial tissue, and SF. We consider these proteins within the context of the pathways with which they have previously been associated, in order to provide a synopsis of their potential biologic significance (Table 3).

**Table 3:** Pathway analysis of select proteins identified from synovial fluid

Serine protease inhibitors	Cartilage metabolism	Collagen metabolism cytoskeletal proteins	Inflammation	Immunologic cascade	Oxidative stress	Apoptosis
AT III	Fibronectin	Collagen V1	Fibrinogen	CLIP	Aflamin	Fibrinogen
C1 inhibitor	IGFBP	α_1_-Antitrypsin	Kinninogen	Complement C3/C4/C6/C8	Paraoxonase	Vimentin
PEDF	Clustrin	Tetranectin	Clusterin	Factor H and factor I	α_1_-Proteinase inhibitor	Apolipoprotein A
α_1_-Antitrypsin	PEDF	Fibronectin	Tetranectin	Fibronectin	Clustrin	H_4 _inhibitor
α_1_-Antichymotrypsin	Pregnancy zone protein	Gelsolin	α_1_-Acid glycoprotein	Fibrinogen	S100	
Kinninogen	Albumin	SHAP	S100	Immuglobulin J polypeptide		
Pregnancy zone protein	CILP	Lumican	PEDF			
C1q (with C1s and C1r)	Cartilage acidic protein	Vimentin	Apolipoprotein A/E			
		COMP	Complement C3/C4/C6/C8			
			Factors H and I			
			Carboxypeptidase			

### Serine protease inhibitors

We identified numerous serine protease inhibitors in the SF of both healthy individuals and patients with diseasepatients (Table 3). The abundance and large number of species of serine proteinase inhibitors is consistent with the importance of the diverse and highly regulated functions of serine proteinases in joint function. Included among the host of physiologic processes in diarthrodial joints regulated by these species are regulation of matrix metalloproteinases (MMPs), aggrecanase, plasmin, tissue mitogens and angiogenesis activity, as well as inhibition of inflammatory leukocyte proteases such as neutrophil elastase and regulation of fibroblast mitogen binding to extracellular matrix [32-42]. Numerous lines of evidence demonstrate that synovial lining and cartilage extracellular matrix undergo active remodeling with joint homeostasis resulting from a delicate balance between matrix degradation, matrix synthesis, and matrix assembly [43]. The importance of this remodeling has been underscored by oncology trials of MMP inhibitors, whose side effects included a progressive polyarthritis with joint pain and stiffness [44-49]. Because the regulation and biologic function of a number of these serine proteinase inhibitors remains incompletely defined, our analyses provide further rationale for their continued study.

### Inflammatory cascades and response to oxidative stress

Oxidative damage and activation of mitogen-activated protein kinases have been reported to be involved in the pathogenesis of OA; our studies identify proteins implicated in these pathways as high-abundance species in SF. S100 activates the receptor for advanced glycation end-products (RAGE) [50,51]. Among the RAGE-stimulated mitogen-activated protein kinase downstream signaling cascades is the increased activity of nuclear factor-κB, which results in increased expression of MMPs and inflammatory mediators [52-55]. Afamin was recently identified as a novel vitamin E binding protein [56]. Vitamin E confers protection from oxidative damage by scavenging reactive oxygen and nitrogen species [57]. Clusterin is produced in numerous tissues during tissue injury or in disease states, and has also been shown to be produced by normal and arthritic chondrocytes [58]. It has numerous proposed functions, including modulation of apoptosis by inhibition of Bax [59]. *In situ *hybridization demonstrates upregulation of clusterin mRNA after exposure of chondrocytes to oxidative stress, and may represent another pathway by which chondrocytes protect themselves from reactive oxygen and nitrogen species [58]. Paraoxonase 1 is another antioxidant protein whose activity probably mirrors the actvities of the other antioxidants identified in the study. The presence of high concentrations of these species in healthy SF suggests that protection from oxidative stress is of particular importance in the avascular cartilage and highly specialized tissue of the joint lining.

The kallikrein-kinin system has been proposed to play a significant role in the inflammatory processes that underlie OA [60,61]. Kallikrein cleaves high-molecular-weight kininogen to yield bradykinin, a potent β_2 _agonist on endothelial cells, resulting in the release of prostacyclin and nitric oxide as well as increased vascular permeability via opening of endothelial cell tight junctions and relaxing of smooth muscle [62-64]. We identified two elements of this system, namely kininogen-1 and N-carboxypeptidase, which is a zinc metalloprotease that degrades bradykinin and anaphylactic peptides of the complement system [65]. These observations are congruent with previous work showing that SF contains all of the components needed to generate kinins [66]. It is possible that disequilibrium between the rate of formation and breakdown of kinins results in the inflammation, joint pain, and swelling that are seen in patients with arthritis.

Our analyses identify members of the potently proinflammatory complement cascade, including components C1, C3, C4, C6 and C8, as well as complement inhibitory proteins factors H and I. Although blood (via ultrafiltration) could deliver complement found in SF, numerous groups have demonstrated complement component production by synovial tissue cells [67-71]. These observations raise the possibility that synovial tissue generates these abundant protein species locally. Functionally, the complement cascade is implicated in innate immunologic defense of the avascular cartilage and SF as well as in the pathophysiology of both OA and rheumatoid arthritis [68,70,72-76].

### Extracellular matrix and cartilage metabolism

Numerous extracellular matrix and cartilage metabolism proteins also comprise a significant fraction of abundant soluble proteins in SF. Collagen type VI (a minor species that is found in hyaline cartilage), cartilage oligomatrix protein (a noncollagenous cartilage glycoprotein) and lumican (a member of the small leucine-rich proteoglycans that bind collagen and cartilage intermediate layer protein) are all constituents of either cartilage or synovial tissue extracellular matrix [77-82]. Their presence in high abundance within healthy SF underscores the highly active tissue repair and remodeling that is present in joint tissues. Other proteins associated with cartilage physiology that are present in high abundance in SF include proteoglycan 4 (a lubricating glycoprotein that is homologous to lubricin) and insulin-like growth factor (IGF)-binding proteins (which regulate the activity of the anabolic protein IGF-I). It is noteworthy that IGF-I is one of the most important trophic factors for cartilage [42,83-85].

Interestingly, our studies identify a number of protein species that have not previously been appreciated as abundant components of SF. Demonstrating expression of hemopexin, tetranectin, inter-α-trypsin inhibitor, histidine-rich glycoprotein, gelsolin, vimentin, and numerous other protein species (Tables 1 and 3) suggests contributions by these classes of protein to SF function. Further analyses of these species promises to provide novel insights into SF physiology in health and disease.

Finally, these pilot data also suggest that differentially expressed abundant protein species in SF could be used as biomarkers for diagnosis and monitoring of therapeutic responses in OA. The ability of these candidate biomarkers to distinguish OA patients from normal individuals adequately will require validation in larger independent cohorts of patients.

### Limitations

Although our studies identify a large number of abundant proteins, a number of anticipated proteins are absent from our list. A striking example is lubricin, a protein whose lubricating properties are critical for both cartilage and synovial lining physiology [11]. Lubricin is present at 200 μg/ml in healthy SF [10,11]. Absence of this protein in our studies suggests that our level of sensitivity is less than 200 μg/ml, or it could represent a technical limitation of our approach. Lubricin has a Mr in excess of 200 kDa, and penetration of large proteins into the primary PAGE separation technique may limit sensitivity [11]. Our results must be interpreted in light of both of these technical limitations.

Articular cartilage matrix undergoes many changes to its structure, molecular configuration, and mechanical properties with age, including surface fibrillations, increased collagen crosslinking, and alterations in proteoglycan structure [86]. Prevalence studies have shown that after age 40 years the incidence of OA increases with every passing decade [86-88]. In an attempt to minimize confounding variables with regard to the analysis of SF from patients with subclinical and preradiographic OA, the control group for this study was chosen from volunteers that were younger than 30 years old. Implicit in this design was lack of age-matched control individuals, because most patients with early and late OA are older than 40 years. In addition, the early OA cohort did not control for patients with inner-third meniscal tears who did not have OA. Finally, the disease-specific performance of these candidate biomarkers was not studied, or were these biomarkers tested against patient populations with varying age, sex, race, or disease etiology (traumatic, infectious, and so on).

Our method of obtaining SF necessitated penetration of the articular space using an 18-gauge needle, a process with obligatory passage through skin and subcutaneous tissue. Our method identified skin-specific keratin species within the abundant proteins in SF. Knowing that these species could only derive from skin, we removed these proteins from our subsequent analyses. The extent of contamination by other skin constituents in our analyses remains undefined.

## Conclusion

Our analyses demonstrate no disease duration-dependent differences in abundant protein composition of SF in OA, and we clearly identify two previously unappreciated distinct subsets of protein profiles in this disease cohort. Additionally, our findings identify novel abundant protein species in healthy SF whose functional contribution to SF physiology was not previously recognized. Finally, these pilot data also suggest that differentially expressed abundant protein species in SF could be used as biomarkers for diagnosis and monitoring of therapeutic responses in OA. The ability of these candidate biomarkers to distinguish OA patients from normal individuals adequately will require validation in larger independent cohorts of patients.

## Abbreviations

IGF = insulin-like growth factor; LC-MS/MS = liquid chromatography with tandem mass spectrometry; MMP = matrix metallproteinase; OA = osteoarthritis; PCA = principal component analysis; RAGE = receptor for advanced glycation end-products; SF = synovial fluid.

## Competing interests

The authors declare that they have no competing interests.

## Authors' contributions

RG was the principle investigator for this study and oversaw or executed the sample acquisition, data analysis, and drafting of the manuscript. DS assisted in the study design and coordination. AK, MC, and DS participated in the analysis of data. BK prepared the samples and processed them using the mass spectrometer. All authors read and approved the final manuscript.
